# Vesicle-mediated transport-related genes are prognostic predictors and are associated with tumor immunity in lung adenocarcinoma

**DOI:** 10.3389/fimmu.2022.1034992

**Published:** 2022-11-29

**Authors:** Changrui Qian, Zewei Jiang, Tong Zhou, Tao Wu, Yi Zhang, Ju Huang, Jinglin Ouyang, Zhixiong Dong, Guang Wu, Jiawei Cao

**Affiliations:** ^1^ Key Laboratory of Laboratory Medicine, Ministry of Education, School of Laboratory Medicine and Life Sciences, Wenzhou Medical University, Wenzhou, China; ^2^ School of Basic Medical Sciences, Wenzhou Medical University, Wenzhou, China; ^3^ Department of Ultrasound Medicine, The Second Affiliated Hospital, Hengyang Medical School, University of South China, Hengyang, China

**Keywords:** lung adenocarcinoma, vesicle-mediated transport-related genes, prognostic signature, tumor immunity, drug sensitivity

## Abstract

**Background:**

Globally, lung adenocarcinoma (LUAD) is the leading cause of cancer-related deaths. It is a progressive disorder that arises from multiple genetic and environmental factors. Dysregulated expression of vesicle-mediated transport-related genes (VMTRGs) have been reported in several cancers. However, the prognostic significance of VMTRGs in LUAD has yet to be established.

**Methods:**

The VMTRG profiling data for 482 LUAD patients and 59 normal controls were downloaded from The Cancer Genome Altas (TCGA). Univariate Cox regression and Least Absolute Shrinkage and Selection Operator (LASSO) regression analyses were performed to construct and optimize the risk model. Several GEO datasets were used to validate the risk model. The roles of these genes were investigated *via* the Kyoto Encyclopedia of Genes and Genomes (KEGG) and gene ontology (GO) enrichment analyses. Differences in immune cell infiltrations between risk groups were evaluated using five algorithms. “pRRophetic” was used to investigate anti-cancer drug sensitivities in two groups. Expression of these five genes in LUAD samples and adjacent normal tissues were evaluated by qRT-PCR. Colony formation and wound healing assays were performed to assess the significance of CNIH1 and AP3S1 in LUAD cells.

**Results:**

We identified 85 prognosis-associated VMTRGs that could be constructed a risk model for LUAD patients, indicating their potential importance in LUAD development. The risk model including the five VMTRGs (CNIH1, KIF20A, GALNT2, GRIA1, and AP3S1) was associated with clinical outcomes. Tumor stage and risk score were found to be independent prognostic factors for LUAD patients. The five VMTRGs were also correlated with activation of the Notch and p53 signaling pathways. The risk model was significantly associated with immune responses and with high-level expression of immune checkpoints. High-risk group patients were more sensitive to several chemotherapeutic drugs and Lapatinib. Furthermore, CNIH1 and AP3S1 promoted LUAD cell growth and migration *in vitro*.

**Conclusion:**

We constructed a VMTRG-based risk model for effective prediction of prognostic outcomes for LUAD patients. The risk model was associated with immune infiltration levels. These five hub genes are potential targets for immune therapy combined with chemotherapy in LUAD.

## Introduction

Globally, lung cancer is among the most common and the leading cause of cancer-related deaths, accounting for 1.8 million deaths (18%) annually (1). Lung adenocarcinoma (LUAD) is the major histological subtype of lung cancer, accounting for about 40% of lung cancer cases (2). Although there have been advances in surgical treatments, neoadjuvant therapies, and immunotherapies, prognostic outcomes for LUAD patients are still unsatisfactory (3, 4). Therefore, there is an urgent need to identify reliable biomarkers to predict the outcomes of LUAD patients.

Vesicle transport mediates basic communication between different compartments in a cell and with the environment. Vesicle-mediated transport, which occurs from within the cell *via* ER and Golgi transport, also plays a role in scavenger receptor-mediated endocytosis (5). The proteins involved in these processes include transmembrane emp24 domain (TMED) family proteins, kinesin superfamily (KIF) proteins, coat protein complex (COP) I proteins, COP II proteins, and Rab family proteins among others (6–8). The TMED family proteins, consisting of type I single-pass transmembrane proteins, are found in all eukaryotes (9). The KIF members mediate all microtubule plus end-directed vesicle transport (10). However, abnormal expression of certain vesicle-mediated transport-related genes are involved in cancer development. TMED3 is involved in vesicle transport and innate immune signal transmission (11). Yu et al. reported that TMED3 is overexpressed in lung squamous cell carcinoma (LUSC) and promotes the progression as well as the development of LUSC by regulating EZR (12). KIF21B acts as an oncogene in LUSC (13). Although the significance of several vesicle-mediated transport related genes in cancers has been reported, the importance of these genes in LUAD should be conclusively explored.

Immune infiltration within the tumor environment (TME) is essential in immune responses, which affects tumor progression and survival outcomes (14–16). The impact of immune checkpoint inhibitors (ICI) in the tumor environment and anti-tumor immunity of LUAD patients has been reported. Vesicles are involved in immune regulation. THADA depletion disrupts interactions between immune checkpoint PD-L1 and Sec24A (a module on the COP II trafficking vesicle), and enhances T cell-mediated cytotoxicity (17). Therefore, the impact of VMTRGs on tumor immunity should be assessed.

We established a vesicle-mediated transport-related genes (VMTRGs) signature for prognostic prediction of LUAD patients and evaluated its association with immune infiltration. The five hub genes (CNIH1, KIF20A, GLANT2, GRIA1, and AP3S1) in the risk model are potential prognostic markers and therapeutic targets for LUAD patients. The risk model has the potential for achieving individualized treatment of LUAD patients.

## Materials and methods

### Study population and data preprocessing

mRNA sequencing (FPKM value) and clinical data for 482 LUAD samples and 59 normal samples were downloaded from The Cancer Genome Atlas (TCGA) (https://portal.gdc.cancer.gov/) database (18). Validation cohorts with full expression profile data and prognostic data (Microarray GPL570: GSE31210, GSE50081, GSE37745; GPL13497: GSE115002; RNA-seq: GSE140343) were downloaded from the GEO (https://www.ncbi.nlm.nih.gov/gds) database (19–21). The 724 VMTRGs were obtained from the Reactome gene set: REACTOME_VESICLE_MEDIATED_TRANSPORT (**Table S2**). For TCGA, FPKM values were transformed into TPM values for further analysis. Twenty-Eight patients with locally LUAD were recruited from the of the Affiliated First Hospital of Wenzhou Medical University (Wenzhou, China) with informed consents, as approved by the Research Ethics Committee. The accession number is 2019-ky-50.

### Cell lines

Lung adenocarcinoma cell lines A549, PC-9, H322, HCC827, H1299, and HEK293T17 were obtained from the American Type Culture Collection (ATCC, Maryland, USA). The normal bronchus epithelial cell line Beas-2B was a gift from Dr Zhixiong Dong, Wenzhou Medical University. The A549 and PC-9 cells were cultured in DMEM/F12 medium (Gibco, California, USA) with 10% fetal bovine serum (Lonsera, Australia), supplemented with 1% penicillin/streptomycin (Beyotime Biotechnology, Jiangsu, China). H322, HCC827, and H1299 cells were cultured in RPMI1640 medium (Gibco) with 10% fetal bovine serum, supplemented with 1% penicillin/streptomycin. Beas-2B and HEK293T17 cells were cultured in DMEM medium (Gibco) with 5% fetal bovine serum, supplemented with 1% penicillin/streptomycin.

### Plasmids

CNIH1-shRNA1 and shRNA2, AP3S1-shRNA1 and shRNA2 (sequences from sigma are shown in **Supplement Table 4**) were subcloned into *Eco*RI and *Age*I digested pLKO.1 vector. All generated plasmids were verified by DNA sequencing (Qingke, Hangzhou, China).

### Lentivirus production and viral infection

The production of lentiviruses and viral infections of cells were performed previously as described (22).

### RNA isolation and qRT-PCR

Total RNAs were isolated from LUAD samples and cell lines using TRIZOL reagent (Invitrogen) according to the manufacturer’s instructions. cDNA was synthesized from 1 μg of purified RNA using Hiscript II Q RT SuperMix (Vazyme, Jiangsu, China) and subjected to quantitative real-time PCR (qRT-PCR) for analysis of relative mRNA of specific genes using ChamQ Universal SYBR qPCR Master Mix (Vazyme). The primers used in this study are shown in the **Supplement Table 4**.

### Colony formation assay

Lung adenocarcinoma cells were cultured in 24-well tissue culture plates for a week to form colonies, fixed in 10% neutral formalin, stained with 0.5% crystal violet solution, and supplemented with 10% acetic acid to extract the dye. Absorbance was measured using a Varioskan flash microplate at 540 nm.

### Wound healing assay

For the wound-healing assay, cells were seeded in 24-well plates. Cells were scratched with a sterile tip perpendicular to the previously painted line. Scratch wounds were imaged using a light microscope after which cell migration was measured at indicated time points of 0 and 24 h.

### Establishment of consistent clustering based on VMTRGs

Univariate Cox regression analysis was performed using the “coxph” function of “survival” in R to identify prognosis-related genes. Eighty-five candidate prognostic genes were screened from among the 724 VMTRGs genes. Expression data for the 85 prognosis-related VMTRGs were used to cluster the LUAD patients into two molecular subtypes using “ConsensusClusterPlus” in R (V 1.56; parameters: reps = 100, pItem = 0:8, clusterAlg = “pam”, pFeature = 1, distance = “Pearson”).

### Construction and validation of a VMTRG-based prognostic signature for LUAD cohorts

Based on the TCGA database, 85 prognostic candidate VMTRGs were screened *via* least absolute shrinkage and selection operator (LASSO) Cox regression analyses to reduce redundancy and avoid model overfitting (23). Five genes were finally selected to construct a prognostic risk score model for predicting the OS of LUAD patients. The risk score formula was:


$$Riskscore\;=\sum_{1}^{i}Coefi*ExpGenei$$

$$\begin{array}{c} Risk\;score=\;0.010428\times CNIH1\;+\;0.02806\times KIF20A\\ \;\;\;\;\;\;+\;0.10947\times GALNT2\;-\;0.01125\times GRIA1\\ \;\;\;\;\;\;+\;0.05029\;\times AP3S1. \end{array}$$

For the TCGA dataset, LUAD patients were assigned into high- and low-risk groups based on median risk score for the LUAD cohort sample. The feasibility of the model was validated using the aforementioned 3 datasets from the GEO database. The Kaplan-Meier (K-M) method was used to assess differences in survival outcomes between the high- and low-risk groups. To establish the independence of the VMTRGs-based signature as a predictor, univariate and multivariate Cox regression analyses were performed using “survival” in R to analyze risk scores and other clinical data, including gender, age, and tumor stage.

### Construction and evaluation of nomograms for LUAD patients

Analysis of the nomogram, including those factors that were distinctly associated with OS in TCGA-LUAD patients in multivariate analysis, was performed using “rms” in R. Differences between actual and nomogram predicted OS probabilities were estimated using the calibration plot.

### Immune infiltration analysis and assessment of drug sensitivity

Immune score, stromal score, estimate score and tumor purity fraction were calculated using the ESTIMATE algorithm “estimate” in R (24). Correlations between the risk score and immune cell infiltration levels were analyzed using CIBERSORT, XCELL, EPIC, TIMER, QUANTISEQ and MCPCOUNTER algorithms in TIMER2.0 (https://cistrome.shinyapps.io/timer/). Single sample GSEA (ssGSEA) was performed using the “GSVA” package. An inhibitory concentration of 50% was determined as the IC50. The IC50s for LUAD patients from the TCGA dataset were calculated using “pRRophetic” in R.

### Functional enrichment analysis

Differential gene expression analyses between high- and low-risk groups were performed using “DESeq2”. “ClusterProfler” in R was used for GO and KEGG enrichment analysis of differentially expressed genes. Gene set enrichment analysis was performed using GSEA v4.0 at a significance threshold of FDR<0.25 and *p*<0.05.

### Statistical analysis

All R packages were executed using the R Studio software (version 4.0.5). ‘ggplot2’ in R was used to map the volcano plot and heatmap. Correlations were determined by Pearson correlation analyses. The log-rank test was used to compare the Kaplan-Meier survival curves. The ROC analysis was performed using ‘Proc’ in R. Statistical significances were established at ****p<0.0001, ***p<0.001, **p<0.01, *p*<0.05, and ns, *p*>=0.05. All experiments were repeated at least three times.

## Results

### The VMTRGs-based prognostic model for LUAD

**Figure 1:**
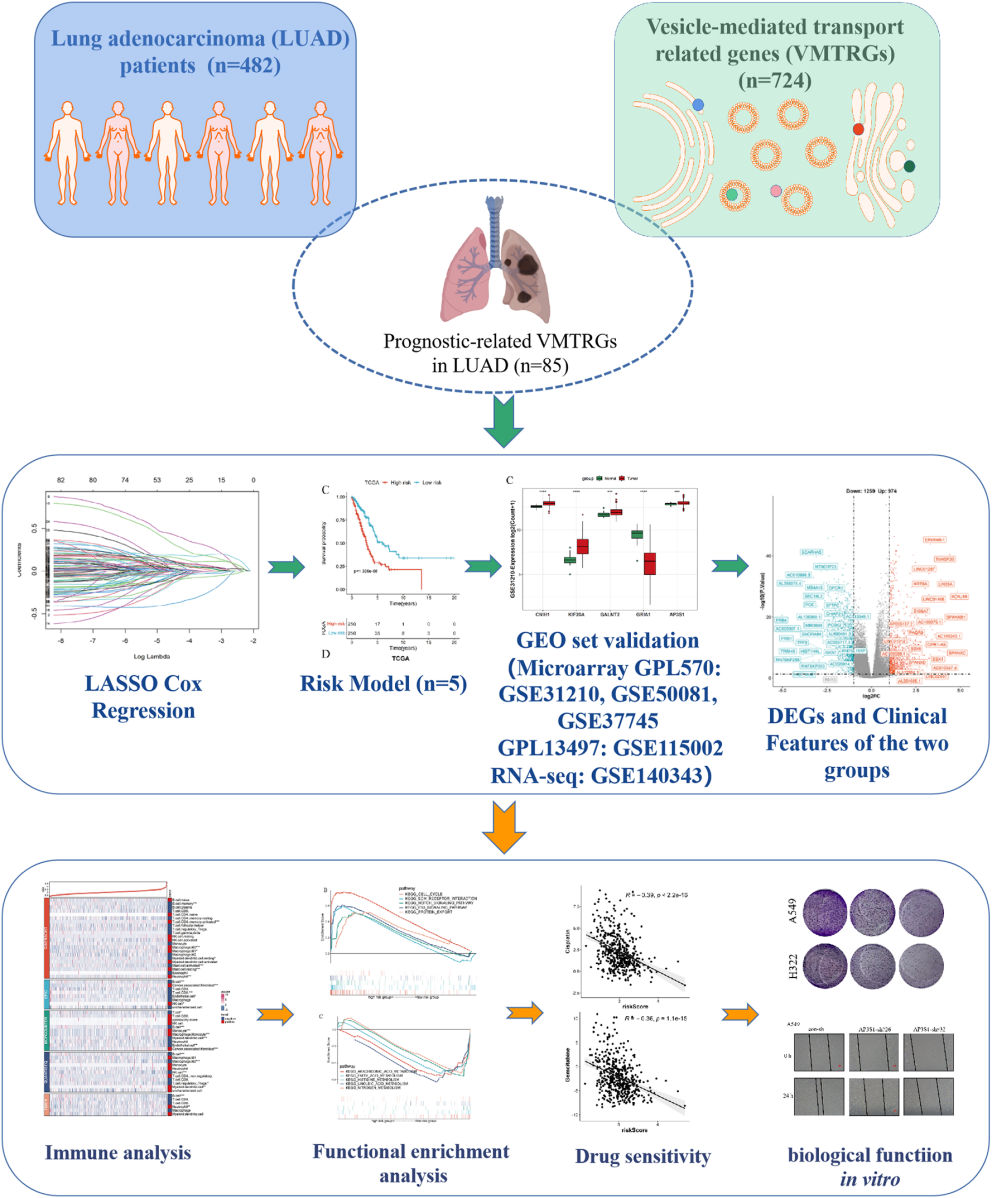
Study flowchart. The 724 LUAD-VMTRGs were first identified from the TCGA dataset after which 85 candidate prognosis-related genes were screened from the 724 VMTRGs. A 5-VMTRGs-based signature was constructed by LASSO analysis. To determine the prognostic value of the signature, we analyzed survival and ROC curve were performed. Finally, a risk model was constructed by combining genetic characteristics with clinical variables.

**Figure 2:**
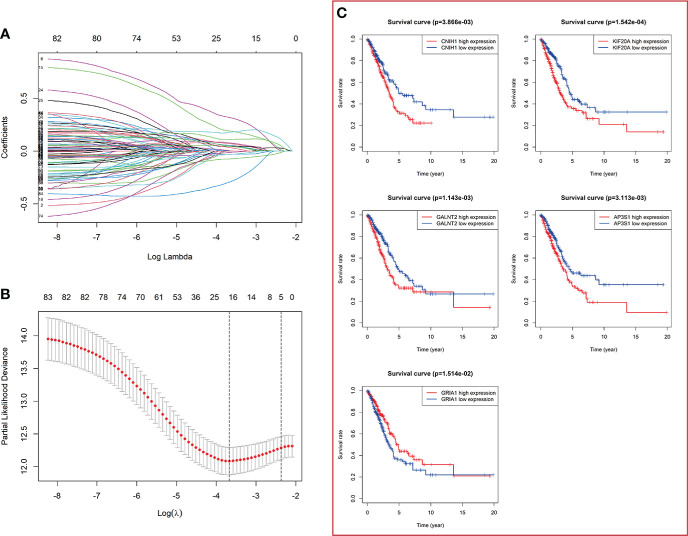
Construction of a vesicle-mediated transport-related genes prognostic model. **(A)** LASSO coefficient spectrum of 85 vesicle-mediated transport-related genes. Each curve represents a gene. **(B)** Cross-validation of adjustment parameter selection in a proportional hazards model. **(C)** The OS from the TCGA-LUAD dataset in relation to genes of constructed prognostic model including *CNIH1*, *KIF20A*, *GALNT2*, *GRIA1*, and *AP3S1*.

The flowchart of the present study is shown in **Figure 1**. Firstly, totals of 85 candidate prognosis-related genes for LUAD were screened from 724 VMTRGs using univariate Cox regression analysis (**Table S3**). To identify the best candidate genes, LASSO Cox regression was used to establish a VMTRGs-based signature. Then, an optimal prognostic model with five non-zero coefficient genes was constructed (**Figures 2A, B**). A median risk score was calculated using the formula: risk score= 0.010428×CNIH1 +0.02806×KIF20A +0.10947×GALNT2 -0.01125×GRIA1 +0.05029×AP3S1. CNIH1, KIF20A, GALNT2, and AP3S1 were hazard factors, while GRIA1 was protective factor in the model. Elevated levels of CNIH1, KIF20A, GALNT2, and AP3S1 were associated with poor outcomes, while suppressed GRIA1 levels were correlated with shorter survival time (**Figure 2C**).

**Figure 3:**
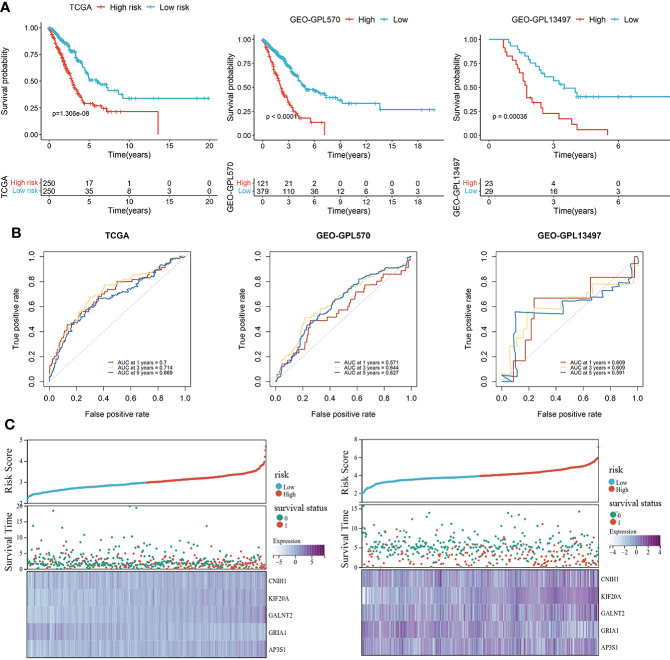
Development and validation of a vesicle-mediated transport-related gene prognostic model. **(A)** Survival analysis of high- and low-risk groups in the TCGA set (left), GEO datasets of GPL570 (middle), and GEO datasets of GPL13497 (right). **(B)** Time-dependent ROC curve analysis of the prediction model in the TCGA and GEO -GPL570 datasets. **(C)** Distribution of expression profiles of 5 hub genes, the risk score, and survival status in TCGA and GEO sets.

The LUAD patients were categorized into high-risk and low-risk groups. Kaplan-Meier survival analysis revealed that prognostic outcomes for the high-risk group were worse than those of the low-risk group in the TCGA set (**Figure 3A**). To evaluate the robustness of the risk model based on the TCGA dataset, multiple microarrays of different platform from the GEO were used for external validation. The high-risk groups were associated with poor prognostic outcomes in the validation sets (**Figure 3A**), consistent with findings from the TCGA training set. The predictive accuracy of the prognostic model was assessed using ROC curves. The AUC for the TCGA cohort at 1-, 3- and 5-years were 0.672, 0.705, and 0.65, respectively (**Figure 3B**). The AUC for the GEO-GPL570 cohorts at 1-, 3-, and 5-years were 0.571, 0.644, and 0.627, respectively, and for GEO-GPL13497 cohorts at 1-. 3-. and 5-years were 0.609, 0.609, and 0.591, respectively (**Figure 3B**). Distribution of expression patterns of five LUAD-VMTRGs, risk scores, survival time, and survival status in TCGA as well as GEO (**Figure 3C**) were also determined. Our findings showed that the LUAD-VMTRG signature was of important prognostic value.

### mRNA and protein expression profiles of five vesicle-mediated transport marker genes in LUAD

**Figure 4:**
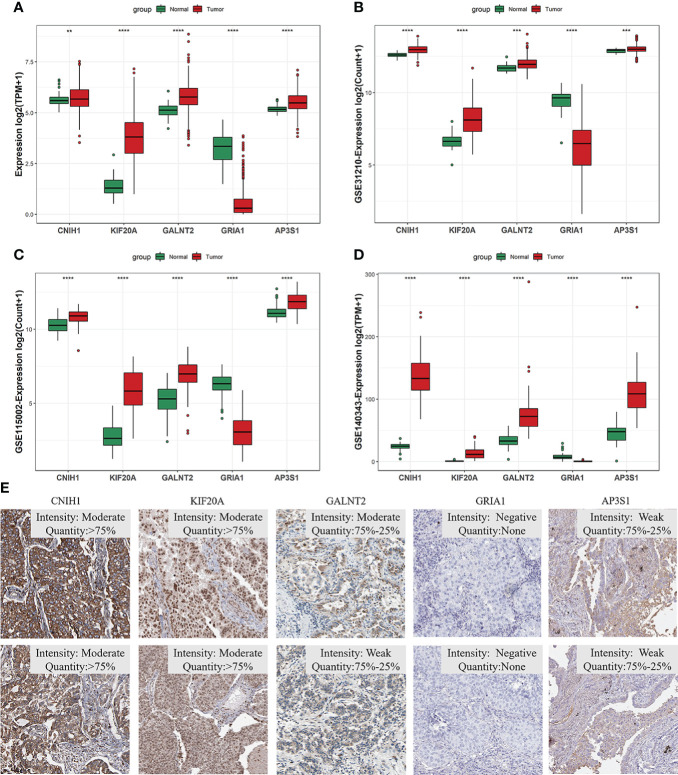
The expression of five hub genes in prognostic model in LUAD. **(A–D)** Differential expression of *CNIH1*, *KIF20A*, *GALNT2*, *GRIA1*, and *AP3S1* between tumor tissues and adjacent tissues in the TCGA database **(A)** and in the three GEO sets [GSE31210 **(B)**, GSE140343 **(C)**, GSE115002 **(D)**]. **(E)** CNIH1, KIF20A, GALNT2, GRIA1, and AP3S1 protein levels in 10 LUAD patients from the HPA database. **p<0.01, ***p<0.001, ****p<0.0001.

Given the five VMTRGs of this risk model were screened based on their prognostic significance, we investigated the expression of each marker gene in LUAD. In the TCGA dataset, mRNA levels of four genes (*CNIH1*, *KIF20A*, *GALNT2*, and *AP3S1*) were highly expressed in tumor tissues, while *GRIA1* levels were downregulated in tumor tissues (**Figure 4A**). mRNA expression of the five genes in the validation datasets (GSE31210, GSE115002, and GSE140343) were comparable. Apart from *GRIA1*, upregulated mRNA levels of *CNIH1*, *KIF20A*, *GALNT2*, and *AP3S1* were observed in tumor tissues in these datasets (**Figures 4B–D**). Expressions of these five genes using our collected LUAD tumor samples. KIF20A and GALNT2 were elevated, whereas GRIA1 expressions were suppressed in tumor tissues, compared with adjacent normal tissues (**Figure S1**). For further verification, protein expression of hub VMTRGs in LUAD were assessed *via* immunohistochemical (IHC) analysis using the Human Protein Atlas (HPA). Protein levels of CNIH1, KIF20A, GALNT2, and AP3S1 were overexpressed in tumor tissues, while GRIA1 levels were not detected in LUAD patients (**Figure 4E**).

### Univariate and multivariate Cox analysis of the VMTRG signature

**Figure 5:**
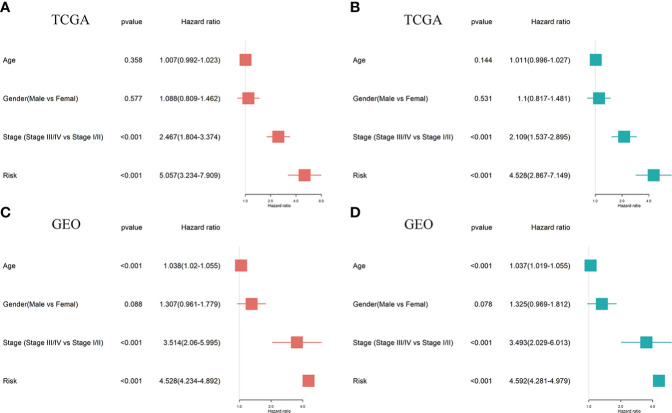
Ability of a risk score and clinical factors to predict the prognosis of LUAD patients. **(A, B)** Univariate **(A)** and multivariate **(B)** Cox regression analyses were used to analyze correlations between OS and other clinical variables, including risk scores in the TCGA database. **(C, D)** Univariate **(C)** and multivariate **(D)** Cox regression analyses were performed to analyze the associations between OS and various clinical variables, including risk scores in the GEO database.

Univariate and multivariate Cox regression analyses were performed to establish if the five-gene signature is an independent predictor for OS of LUAD patients. The patient's clinical data and risk scores are listed in **Supplement Table S1** Univariate Cox regression analysis of TCGA data revealed that tumor stage and VMTRG risk score are independent prognostic variables for LUAD patients (HR=2.467, *p*<0.001 and HR=5.057, *p*<0.001) (**Figure 5A**). Multivariate Cox regression analysis also showed that tumor stage and VMTRG risk score are independent prognostic factors for LUAD patients in the TCGA dataset (HR=2.109, *p*<0.001 and HR=4.528, *p*<0.001) (**Figure 5B**). Then, the GEO sets were used to verify this risk model. Univariate and multivariate Cox analysis also showed that tumor stage (HR=3.514, *p*<0.001 and HR=3.493, *p*<0.001) and VMTRG risk (HR=4.528, *p*<0.001 and HR=4.592, *p*<0.001) are also independent prognostic factors (**Figures 5C, D**).

### Construction and evaluation of the nomogram

**Figure 6:**
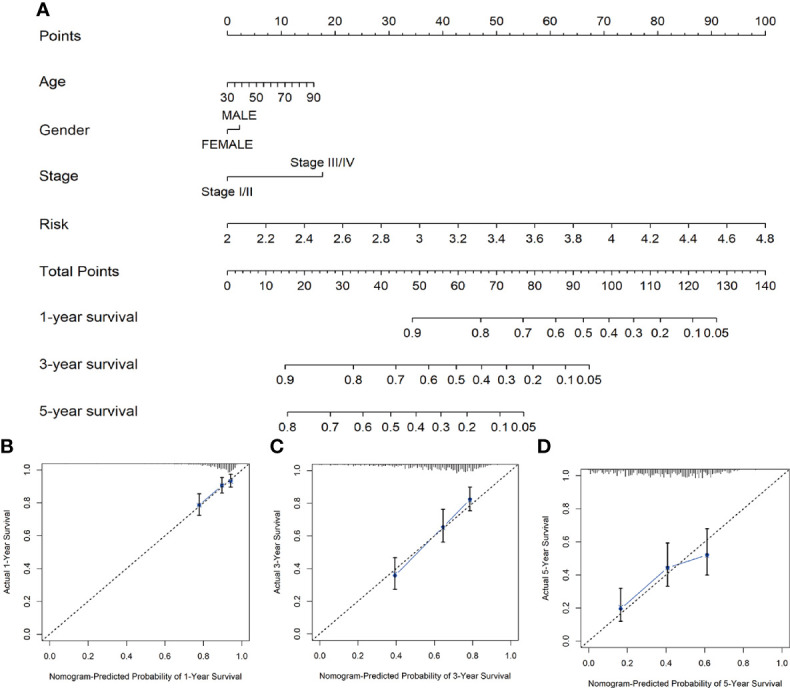
**(A)** Nomogram survival prediction models for predicting the OS of patients with LUAD at 1-, 3-, and 5-years. **(B–D)** Calibration plots of the nomogram at 1- **(B)**, 3- **(C)**, and 5- **(D)** year survival.

We constructed a nomogram composed of VMTRGs and other clinical factors, including stage, age, and sex to further assess their predictive ability for individual OS outcomes. Predictive effects of the nomogram for 1-, 3-, and 5-year OS outcomes in LUAD patients were calculated (**Figure 6A**). The calibration chart revealed that effectiveness of the nomogram was accurate (**Figures 6B–D**). Therefore, the vesicle-mediated transport-related genes are potential prognostic markers for LUAD.

### The immune microenvironment

**Figure 7:**
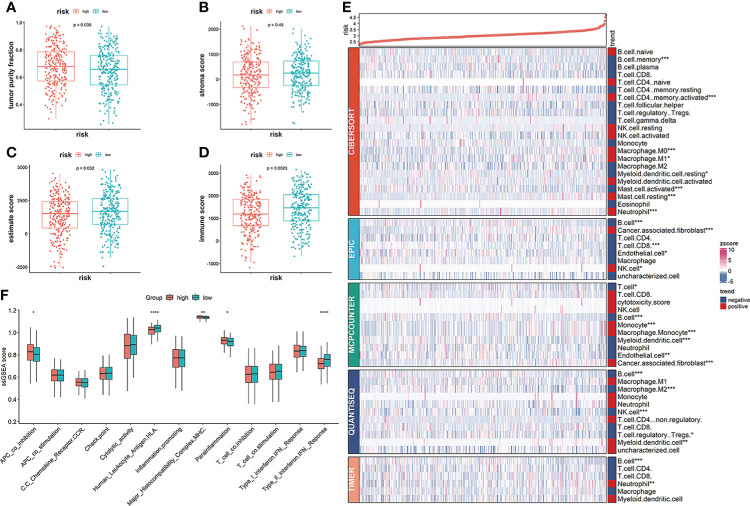
Correlation of vesicle-mediated transport-related gene signature with immune cell infiltrations in the TCGA cohort. **(A, B)** Stromal scores and tumor purity fractions between the risk groups. **(C, D)** Differences in immune and estimate scores between the two risk groups were significant. **(E)** Differences in abundance of immune cell infiltration between the high-risk and low-risk groups. **(F)** ssGSEA scores of immune functions between the high- and low-risk groups. *p<0.05, **p<0.01, ***p<0.001, ****p<0.0001.

**Figure 8:**
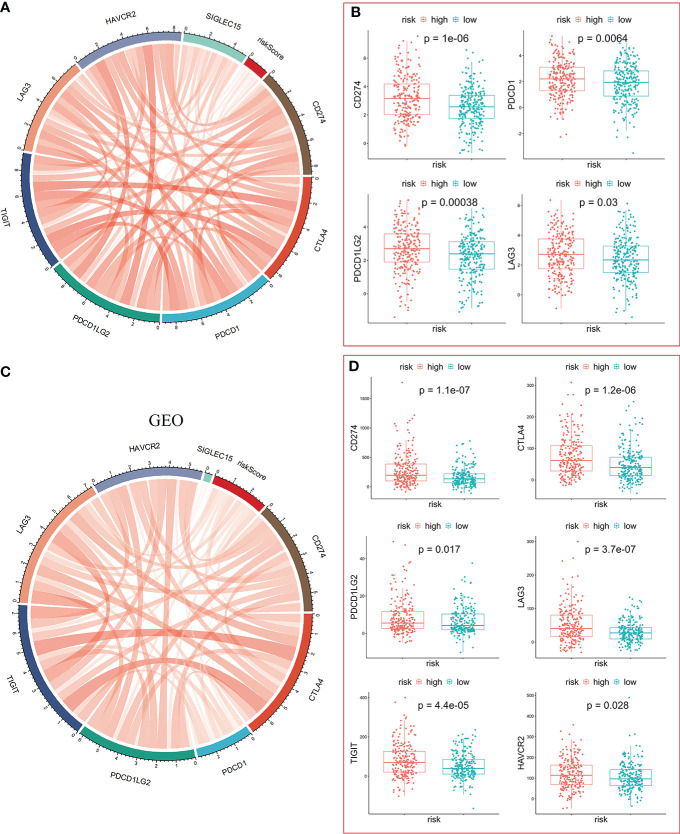
Significance of the risk model in immunotherapy. **(A, C)** The risk score was positively correlated with expression of immune checkpoint molecules in TCGA-LUAD **(A)** and GEO-GPL570-LUAD **(C)**, respectively. **(B)** The mRNA levels of CD274, PDCD1, PDCD1LG2, and LAG3 were higher in the high-risk group. **(D)** The mRNA levels of CD274, CTLA4, PDCP1LG2, LAG3, TIGIT, and HAVCR2 were higher in the high-risk group.

Clinical outcomes for tumor patients are closely correlated with the abundance of tumor immune cells in the tumor microenvironment, making them ideal targets for cancer treatment (25). Therefore, we investigated the association between the risk score and the immune microenvironment. Tumor purity fraction was higher in the high-risk group, compared to the low-risk group (**Figure 7A**). Differences in stromal scores between the two groups were not significant (**Figure 7B**). However, estimate (**Figure 7C**) and immune (**Figure 7D**) scores were significantly lower in the low-risk group. Immunofunctional analysis showed that APC co-inhibition and major histocompatibility complex (MHC) were highly active in the high-risk group, compared with the low-risk group, while cytolytic activity and type II interferon IFN responses were more active in the low-risk group (**Figure 7F**). Correlation between the immune microenvironment and risk scores was further assessed by constructing different immune cell profiles in LUAD samples using the five algorithms (CIBERSORT, EPIC, MCPCOUNTER, QUANTISEQ and TIMER) (**Figures 7E**). There was a high infiltration of cancer-associated fibroblasts as well as CD8+ T cells and a low infiltration of B cells in the patients. The high-risk score was correlated with high expression of immune checkpoints (**Figures 8A, C**), especially CD274, PDCD1, PDCD1LG2 and LAG3 (**Figure 8B**) in TCGA cohort, and with CD274, CTLA4 in GEO sets (**Figure 8D**). Patients in high-risk groups may benefit from immunotherapies, if treated with anti-PD-L1 antibodies. In summary, quantification of vesicle-mediated transport risk score is of great importance for LUAD patients, and may be a new biomarker for clinical treatment.

### Functional enrichment analysis

**Figure 9:**
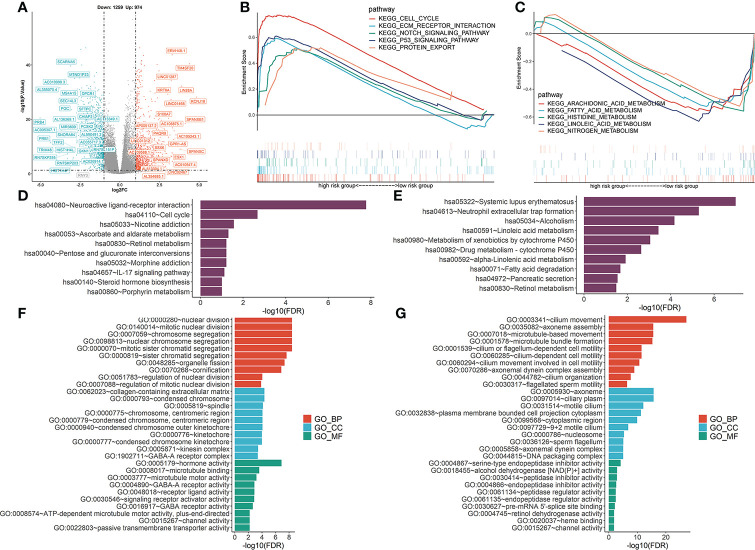
Functional analysis was performed to identify the enriched biological processes. **(A)** Volcano map of differentially expressed vesicle-mediated transport-related genes in TCGA-LUAD cohort. **(B, C)** GSEA analysis of high- **(B)** and low- **(C)** risk groups. NES: normalized enrichment scores. **(D, E)** KEGG analysis of high- **(D)** and low- **(E)** risk groups. **(F, G)** The GO enrichment analyses of high- **(F)** and low- **(G)** risk groups. BP, biological processes; CC, cellular components; MF, molecular functions.

To determine the potential molecular characteristics of the two risk groups, differentially expressed genes (DEGs) of the high- and low-risk groups were screened. The volcano map was created to depict DEGs (**Figure 9A**). GSEA pathway analyses of the high-risk group showed that the DEGs were predominantly enriched in the cell cycle, EMC receptor interaction, notch signaling pathway, p53 signaling, and protein export signaling pathway (**Figure 9B**), whereas DEGs in the low-risk group were predominantly enriched in multiple metabolic processes, including arachidonic acid metabolism, fatty acid, histidine metabolism, linoleic acid metabolism, and nitrogen metabolism (**Figure 9C**). KEGG analysis showed that DEGs in the high-risk group were highly enriched in neuroactive ligand-receptor interactions, the IL-17 signaling pathway, and steroid hormone biosynthesis (**Figure 9D**), while drug metabolism-cytochrome P450 and other metabolic pathways were enriched in the low-risk group (**Figure 9E**). Meanwhile, GO functional analysis showed that DEGs in the high-risk group were enriched in nuclear division and chromosomal segregation (**Figure 9F**), while DEGs in the low-risk group were correlated with cilium movement and axoneme assembly (**Figure 9G**). These findings prove the significance of VMTRGs and the potential molecular mechanisms involved in LUAD.

### Responses to anti-cancer therapies

**Figure 10:**
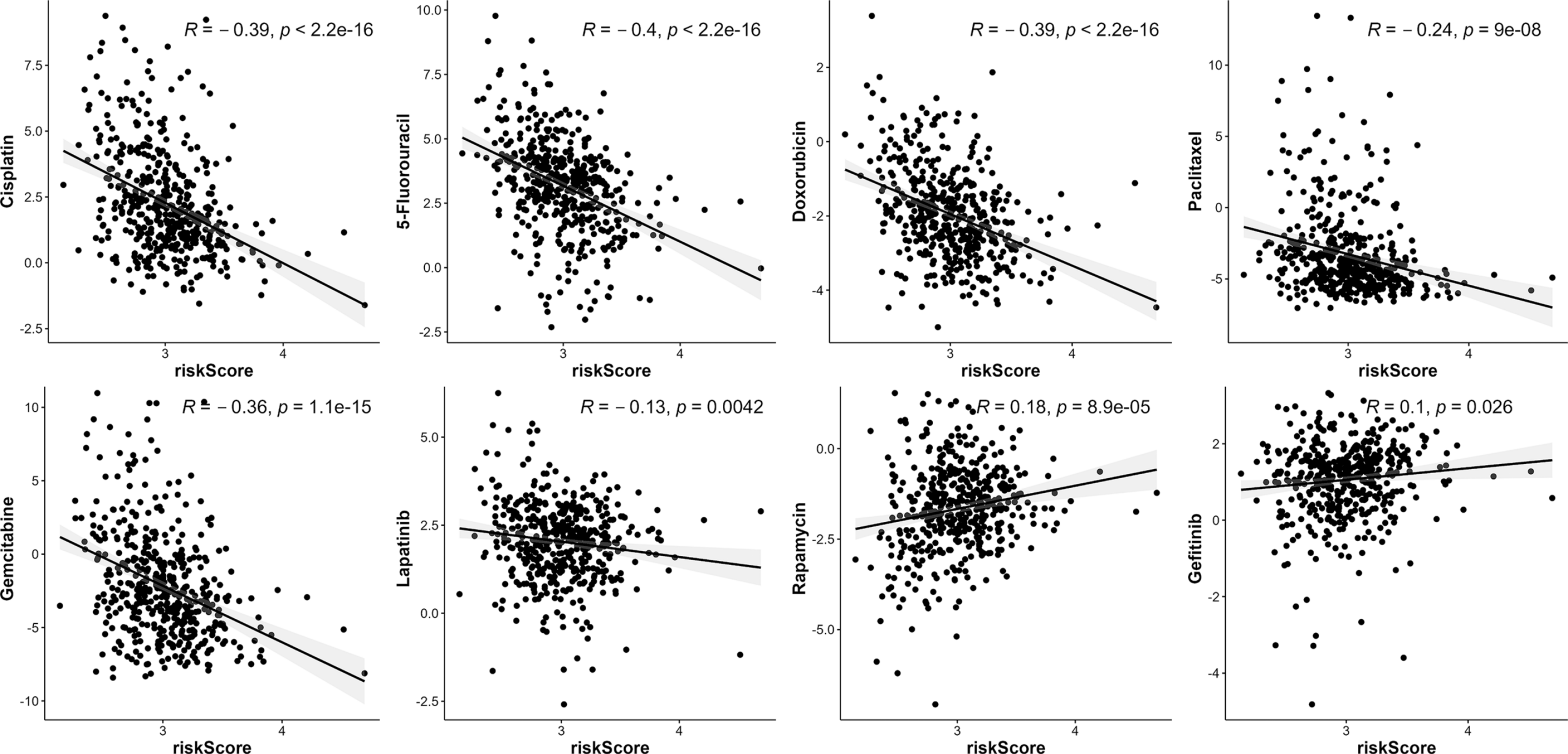
The association between risk score and drug sensitivity. VMTRG risk score was correlated with Cisplatin, 5-Fluorouracil, Doxorubicin, Paclitaxel, Gemcitabine, Lapatinib, Rapamycin, Gefitinib drug sensitivity (IC50) in the TCGA-LUAD.

Given that the risk score was correlated with poor outcomes, we investigated the correlations between anti-cancer drug sensitivity and risk score. In **Figure 10**, high-risk score patients were highly sensitive to certain chemotherapeutic medicines, including Cisplatin, 5-Fluorouracil, Doxorubicin, Paclitaxel, Gemcitabine, as well as the RTK inhibitor (Lapatinib). Moreover, high-risk scores were correlated with high IC50 of Rapamycin and Gefitinib, which indicated that high-risk score patients were resistant to Rapamycin and Gefitinib.

### Biological significances of CNIH1 and AP3S1 in LUAD progression

**Figure 11:**
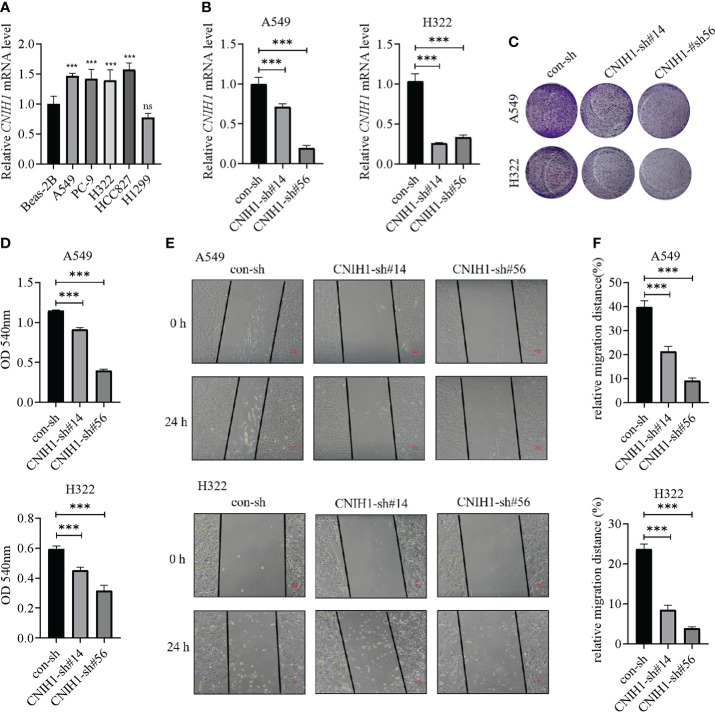
Expression of CNIH1 in LUAD cells and its function. **(A)** CNIH1 mRNA levels in Beas-2B and LUAD cells. **(B)** The mRNA levels of CNIH1 in A549 and H322 LUAD cells after its knockdown. **(C, D)** Colony formation assays are used to explore the functions of CNIH1 in LUAD cells. The representative images were shown in **(C)**. **(E, F)** Knockdown of CNIH1 inhibits LUAD cells migration. The wound healing assay was performed to assess the migration of A549 and H322 cells after CNIH1 knockdown. Representative images were shown in **(E)**. ***p<0.001 ns, no significance. All experiments were repeated at least three times.

**Figure 12:**
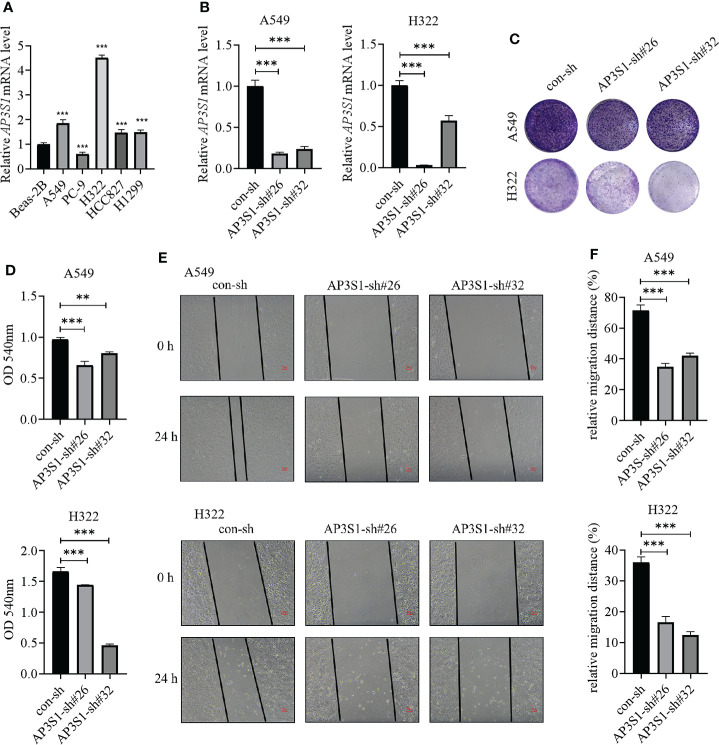
Expressions of AP3S1 in LUAD cells and its function. **(A)** AP3S1 mRNA levels in Beas-2B and LUAD cells. **(B)** The mRNA levels of AP3S1 in A549 and H322 LUAD cells after its knockdown. **(C, D)** Colony formation assays were used to assess the functions of AP3S1 silenced in LUAD cells. Representative images were shown in **(C)**. **(E, F)** Knockdown of AP3S1 inhibits the migration of LUAD cells. The wound healing assay was performed to examine the migration of A549 and H322 cells with AP3S1 expression being silenced. Representative images were shown in **(E)**. **p<0.01, ***p<0.001. All experiments were repeated at least three times.

To assess the roles of these five genes in the risk model in LUAD, various *in vitro* assays were performed. The oncogenic roles of KIF20A and GALNT2 in LUAD have been reported before (26, 27). Since GRIA1 was downregulated in LUAD tumor tissues, CNIH1 and AP3S1 were selected for further studies. First, expressions of CNIH1 and AP3S1 in LUAD cells and in normal bronchus epithelial cells (Beas-2B) were evaluated. In **Figures 11A** and **12A**, mRNA expression of CNIH1 and AP3S1 were elevated in LUAD cells, compared with Beas-2B cells. Moreover, we silenced CNIH1 and AP3S1 expression in A549 and H322 cells (**Figures 11B**, **12B**). Knockdown of CNIH1 and AP3S1 inhibited the growth (**Figures 11C, D**, **12C, D**) and migration (**Figures 11E, F**, **12E, F**) of LUAD cells, implying that CNIH1 and AP3S1 promote the LUAD progression.

## Discussion

Advances in high-throughput sequencing technologies have improved the identification of prognosis-related genes and therapeutic targets (28, 29). However, there is a lack of reliable prognostic biomarkers for LUAD. In eukaryotic cells, vesicle-mediated transport plays a key role in cancer development (9, 30). Several VMTRGs promote the progression of various cancers. Studies on the TMED family of proteins revealed that they have an important impact on cell growth, differentiation, and malignancy. TMED2 is the sole member of the vertebrate TMED β subfamily and exhibits cell type-specific effects in cancer (31, 32). Therefore, VMTRGs are involved in cancer progression.

We established a VMTRGs-based risk model using public databases. Univariate and multivariate Cox analyses showed that stage and risk scores are independent prognostic indicators for LUAD patients. Univariate Cox analyses indicated that five VMTRGs, including *CNIH1*, *KIF20A*, *GALNT2*, *GRIA1*, and *AP3S1* are survival-related genes. These genes were used to establish a five-gene prognostic signature for LUAD patients.

The cornichon protein (CNIH1), a functionally conserved protein family, is involved in selective transport and maturation of the TGFα family (33). Moreover, CNIH1 is predicted to be involved in immune response, where it may affect immune infiltration by transporting the TGFα family protein in LUAD. Mitotic kinesin is involved in chromosome passenger complex (CPC)-mediated cytokinesis. KIF20A can act as a motor required for retrograde RAB6-regulated transport of Golgi membranes and associated vesicles along microtubules (34). In this study, the high-risk group was enriched with “mitotic nuclear division” and “microtubule motor activities” (**Figure 9F**). KIF20A activates the autocrine of androgen receptors, promotes prostate cancer development (35) and is a promising target for cancer immunotherapy. The efficacies of KIF20A-derived peptides combined with Gemcitabine were evaluated in a phase I clinical trial involving advanced pancreatic cancer patients who had previously received chemotherapy and radiotherapy (36). Phase I/II clinical trials of immunotherapy for lung cancer, pancreatic cancer, and cholangiocellular carcinoma using KIF20A-derived short peptides are underway (37)

GALNT2 is an ionotropic glutamate receptor, and in the central nervous system, L-glutamate is an excitatory neurotransmitter at many synapses. Our KEGG analysis showed that the high-risk group was enriched in neuroactive ligand-receptor interactions (**Figure 9D**). GALNT2 expression in LUAD tissues were established to be higher than in normal tissues, and GALNT2 hypomethylation predicted worse overall survival outcomes (38). Moreover, GALNT2 promotes the proliferation, migration, and invasion of LUAD cells (27).

Initial reactions for biosynthesis of O-linked oligosaccharides are catalyzed by GRIA1, followed by the transfer of an N-acetyl-D-galactosamine residue to a serine or threonine residue at the protein receptor. Janez Jazbec et al. reported that GRIA1 gene polymorphisms are a risk factor for asparaginase hypersensitivity in children with acute lymphoblastic leukemia (39). AP3S1, which is part of the AP-3 complex, is correlated with Golgi regions and more peripheral structures. It promotes the budding of vesicles from the Golgi membrane and may directly participate in transport to lysosomes. AP3S1 is overexpressed in most tumors, and its overexpression is correlated with low survival rates (40). These findings were in line with those obtained in this study.

Enrichment analysis showed that the high-risk model was positively correlated with “cell cycle”, “Notch signaling pathway”, “p53 signaling pathway”, and negatively correlated with “arachidonic acid metabolism”, “fatty acid metabolism”, and other metabolic pathways (**Figure 9**). Thus, the five VMTRGs may influence cell cycle regulation in LUAD and drive malignant progressions of LUAD by activating the Notch and p53 signaling pathways.

The high-risk group was associated with low estimate and immune scores. Besides, ssGSEA analysis showed that the high-risk group was correlated with high APC co-inhibition, major histocompatibility complex, and para inflammation, while the low-risk group was associated with human leukocyte antigen (HLA) and type II interferon responses (**Figure 7F**). Furthermore, the high-risk group was positively correlated with cancer-associated fibroblasts (**Figure 7E**). The secretome of CAFs, which are major producers of ECM components and many other secreted factors, directly and indirectly regulate cancer progression and tumor immunity (41). We found a high abundance of CAFs in the TME of the high-risk group, which inhibited immune cell functions, leading to LUAD progression. Findings from CIBERSORT, EPIC, MCPCOUNTER, QUANTSEQ, and TIMER algorithms showed negative correlations between the high-risk group and B cell infiltrations (**Figure 7E**). Plasma cells are differentiated into tumor-associated B cells that then produce tumor-specific antibodies to recognize and react against tumor-associated antigens (42). Low B cell infiltrations in the high-risk group may promote LUAD progression.

The high-risk score was associated with positive expression of immune checkpoints (**Figure 8**), especially CD274 expression, suggesting that high risk LUAD patients may highly benefit from immunotherapy, particularly anti-PD-1 or PD-L1 antibodies. High-risk score patients were more sensitive to several anti-cancer drugs, including Cisplatin, 5-Flu, Doxorubicin, and to the RTK inhibitor, Lapatinib. These five genes may serve as preclinical guidelines for lung adenocarcinoma. Patients with high expression of KIF20A, GALNT2, CNIH1, AP3S1, and low expression of GRIA1 may benefit from combination of immunotherapy and chemotherapy.

Despite our findings, this study has some limitations. The mRNA levels of CNIH1 and AP3S1 detected in our own LUAD samples were not fit the public data, which may result from that expression of these genes in different tumor stages of LUAD maybe different. More LUAD samples should be evaluated further to confirm verify of findings. The significance of the five genes *in vivo.* Moreover, it should be determined how these five VMTRGs affect immune infiltrations and anti-tumor immunity should be determined.

## Conclusions

We developed a vesicle-mediated transport risk model for prognostic prediction of LUAD patients and showed that the vesicle-mediated transport risk score is correlated with immune cell infiltrations. CNIH1 and AP3S1 promoted the growth and migration of LUAD cells *in vitro*. These five hub genes are potential therapeutic targets for individualized treatment of LUAD patients.

## Data availability statement

The datasets presented in this study can be found in online repositories. The names of the repository/repositories and accession number(s) can be found in the article/**Supplementary Material**.

## Ethics statement

The studies involving human participants were reviewed and approved by The Affiliated First Hospital of Wenzhou Medical University (Wenzhou, China). The patients/participants provided their written informed consent to participate in this study.

## Author contributions

CQ, ZJ, TZ, TW, YZ, JH, and JO analyzed and interpreted the data. ZD, GW, and JC conceived and supervised the study. CQ, ZJ, and JC drafted and wrote the manuscript. All authors read and approved the final version of the manuscript for submission.

## Funding

This work was supported by grants from the Zhejiang Provincial Natural Science Foundation of China (No. LY20C080001), the National Natural Science Foundation of China (No. 82272702), the Wenzhou Science and Technology Bureau of China (No. Y2020247), the Provincial Department of Science and Technology, and the Natural Science Foundation of Hunan Province (No. 2022JJ40389), the Wenzhou Medical University Startup Funds, and the Key Discipline of Zhejiang Province in Medical Technology (First Class, Category A).

## Acknowledgments

We thank the participants who provide data in TCGA and GEO databases. Much appreciation goes to the American Editor editing company for editing the language of this manuscript.

## Conflict of interest

The authors declare that the research was conducted in the absence of any commercial or financial relationships that could be construed as a potential conflict of interest.

## Publisher’s note

All claims expressed in this article are solely those of the authors and do not necessarily represent those of their affiliated organizations, or those of the publisher, the editors and the reviewers. Any product that may be evaluated in this article, or claim that may be made by its manufacturer, is not guaranteed or endorsed by the publisher.

## Supplementary material

Supplementary Table 1 Clinical data for all LUAD patients were obtained from the TCGA database.

Supplementary Table 2 Vesicle-mediated transport-related genes.

Supplementary Table 3 Coefficient and p-Value of genes.

Supplementary Table 4 The primers used in this study.

Supplementary Figure 1 Expression of genes in risk model from collected LUAD samples. The mRNA levels of CNIH1, KIF20A, GALNT2, GRIA1, and AP3S1 in 28 LUAD patients from Wenzhou Medical University Affiliated Hospital were determined using RT-qPCR. ****p*<0.001, ns=no significance.

## Abbreviations

AUC, area under the ROC curve; BP, biological processes; CAF, cancer associated fibroblast; CC, cellular components; coefi, coefficient; DEGs, differentially expressed genes; FBS, fetal bovine serum; FDR, false discovery rate; GO, Geno ontology; GSEA, Gene set enrichment analyses; KEGG, Kyoto Encyclopedia of Genes and Genomes; KM, Kaplan-Meier; ICI, immune checkpoint inhibitor; IHC, immunohistochemical; LASSO, Least Absolute Shrinkage and Selection Operator; MF, molecular function; LUAD, lung adenocarcinoma; ssGSEA, single-sample gene set enrichment analysis; qPCR, quantitative real-time PCR; RTK, receptor tyrosine kinase; TCGA, The Cancer Genoma Altas; TME, tumor environment; VMTRG, vesicle mediated transport related gene.
