# Supplementary figures and images for: Vesicle-mediated transport-related genes are prognostic predictors and are associated with tumor immunity in lung adenocarcinoma
Source: Front Immunol. 2022 Nov 29;13:1034992. doi: 10.3389/fimmu.2022.1034992 (PMC9745133; doi:10.3389/fimmu.2022.1034992)

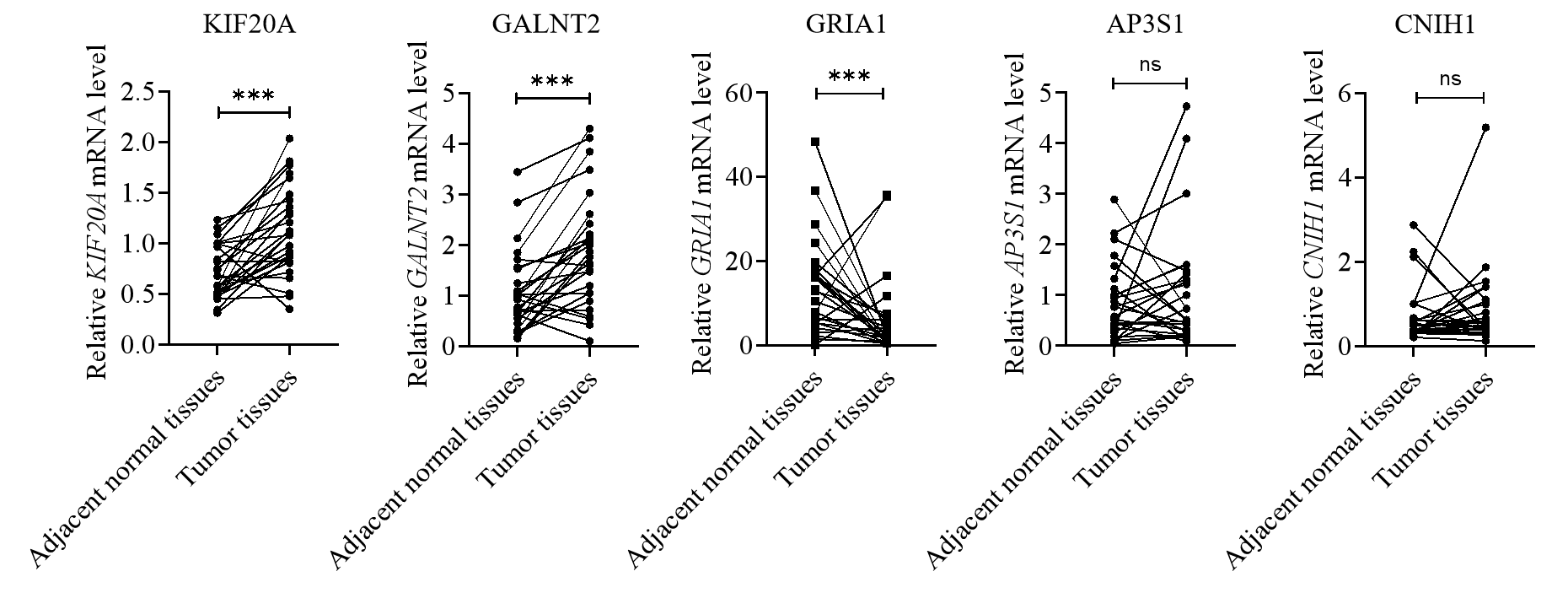

Supplement: Supplementary Figure 1 — Expression of genes in risk model from collected LUAD samples. The mRNA levels of CNIH1, KIF20A, GALNT2, GRIA1, and AP3S1 in 28 LUAD patients from Wenzhou Medical University Affiliated Hospital were determined using RT-qPCR. ***p<0.001, ns=no significance. [file Image_1.tif]
